# Supplementary material for: Hcp of the Type VI Secretion System (T6SS) in Acidovorax citrulli Group II Strain Aac5 Has a Dual Role as a Core Structural Protein and an Effector Protein in Colonization, Growth Ability, Competition, Biofilm Formation, and Ferric Iron Absorption
Source: Int J Mol Sci. 2022 Aug 25;23(17):9632. doi: 10.3390/ijms23179632 (PMC9456162; doi:10.3390/ijms23179632)
Supplement: Supplementary file 1 [file ijms-23-09632-s001.zip › ijms-1788839-supplementary.pdf]

Supplementary Materials

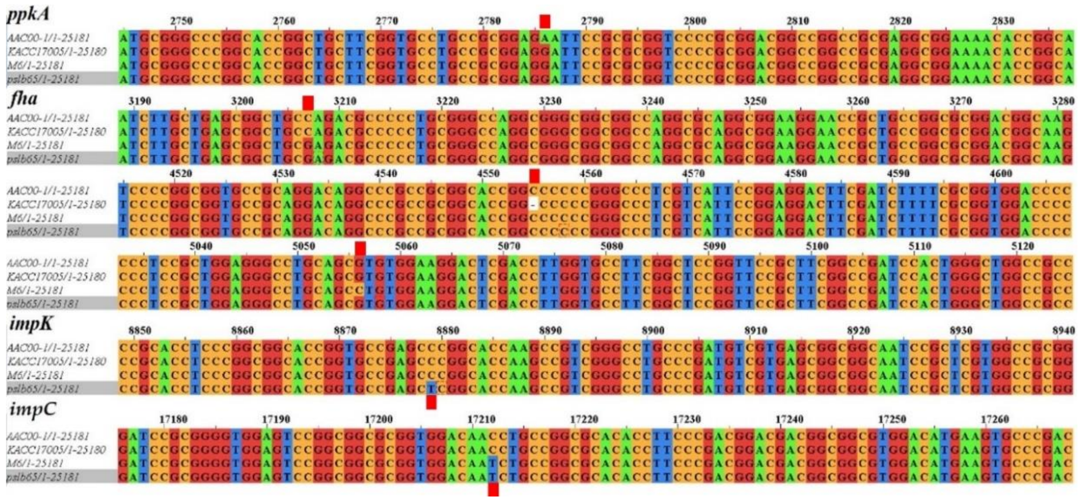

Figure S1: The sequence differences of genes in the T6SS cluster in *Acidovorax citrulli* strain AAC00-1, KACC17005, M6, and pslb65 by Clustal and Jalview.

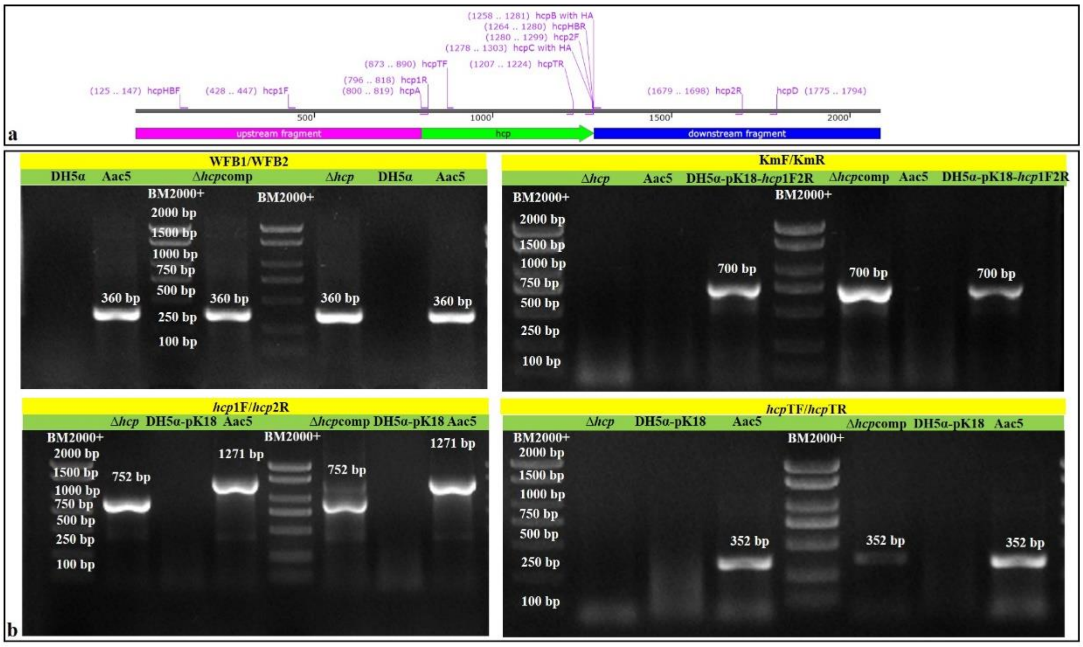

Figure S2: Primer positions and verification of *hcp* deletion mutant strain. (a) Primer positions for Hcp described in this study. The whole open reading frame (ORF) of *hcp* was deleted but not the upstream and downstream fragments. An HA tag was fused to the *hcp* C-terminal before the stop codon by primer *hcp*A, B, C, D; (b) Verification of *hcp* deletion mutant strain by WFB primers (should be positive in Δ*hcp*), Km primers (should be negative Δ*hcp*), *hcp* specific primers (should be negative Δ*hcp*), and *hcp*1F/ *hcp*2R (should be a smaller fragment in Δ*hcp* than wild type strain Aac5).

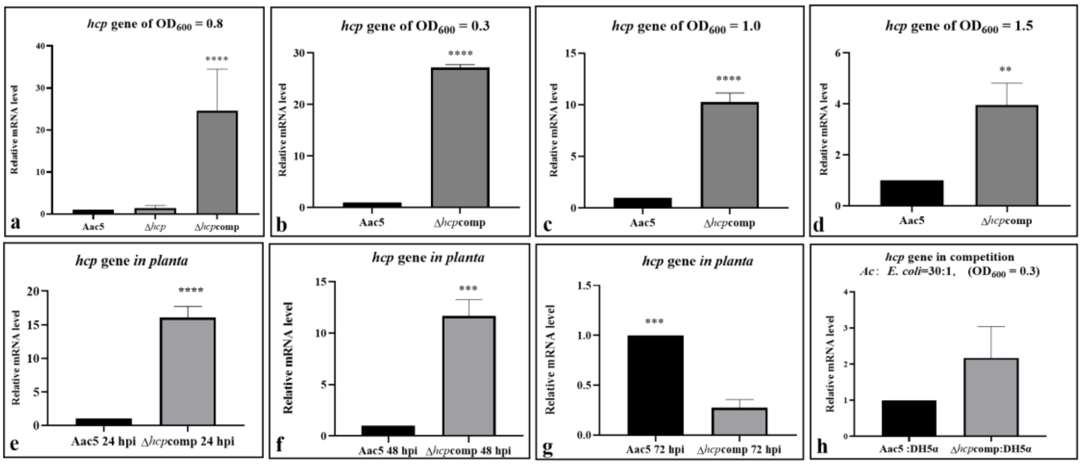

Figure S3: Relative expression of *hcp* gene. (a) Relative expression of *hcp* gene in wild type strain Aac5, Δ*hcp* and complementary strain Δ*hcp*comp when cultured to OD_{600} = 0.8 in medium, (p<0.0001, ****); Relative expression of *hcp* gene in wild type strain Aac5 and complementary strain Δ*hcp*comp of different OD_{600} in KB medium; (b) OD_{600} = 0.3 (p<0.0001, ****), (c) OD_{600}=1.0 (p<0.0001, ****), (d) OD_{600}=1.5 (p<0.01, **); Relative expression of *hcp* gene in wild type strain Aac5 and complementary strain Δ*hcp*comp of different time in planta (e) 24 hpi (p<0.0001, ****), (f) 48 hpi (p<0.001, ***), (g) 72 hpi (p<0.001, ***); (h) Relative expression of *hcp* gene in wild type strain Aac5 and complementary strain Δ*hcp*comp when co-cultured with DH5α at the ratio of *Ac* : DH5α=30:1 for 3 h (There was no significant difference between Aac5 and Δ*hcp*comp).

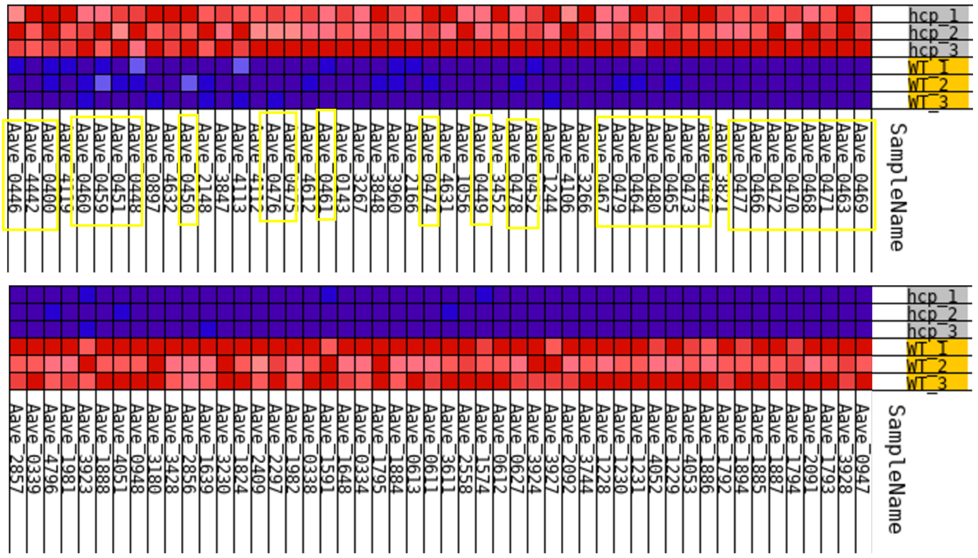

Figure S4: Heat map of differential expression genes between Aac5 and Δ*hcp* at OD_{600} = 1.5 by transcriptome analysis. The differential expression genes associated with T3SS were marked by yellow boxes.

Table S1 Strains and plasmids used in the study

Strains and plasmids	Relevant characteristics	Source
Strain		
Aac5	*Acidovorax citrulli* wild-type group II strain, Amp^R	Yan *et al*., 2013
Δ*hcp*	*hcp* markerless mutant of Aac5, Amp^R	This study
Δ*hcp*comp	Δ*hcp* containing pBBRMCS2 carrying *hcp* with its native promoter; Amp^R; Km^R	This study
Δ*tssm*	*tssm* markerless mutant of Aac5, Amp^R	This study
Δ*hcp*Δ*tssm*	*hcp* and *tssm* markerless mutant of Aac5, Amp^R	This study
Aac5-hcp-HA	*hcp* fused with HA at its C-terminal in Aac5	This study
Δ*hcp*-hcp-HA-pBBR	Δ*hcp* containing pBBRNolac-HA carrying *hcp* with its native promoter; Amp^R; Km^R	This study
Δ*tssm*-hcp-HA	*hcp* fused with HA at its C-terminal in Δ*tssm*	This study
DH5*α*	*supE44 ΔlacU169* (*Φ80/lacZ*ΔM15) *hsdR17 recA1 endA1 gyrA96 thi-1 relA1*	Tiangen
DH5*α*-pRK600	Helper strain in tri-parental mating; Cm^R	Lab collection
DH5*α*-pBBRMCS5	DH5*α* with pBBRMCS5, Gm^R	This study
ATCC27853	*Pseudomonas aeruginosa*	Beijing Zoman Biotechnology
GS115	*Pichia pastoris*	Beijing Zoman Biotechnology
Y2HGold	*Saccharomyces cerevisiae*	Lab collection
pslb65-pBBRMCS5	Group I strain of *A. citrulli* with pBBRMCS5, Gm^R	This study
Plasmid		
pK18mob*sacB*	Suicide vector with a *sacB* gene for mutagenesis; Km^R	Lab collection
pBBRNolac-HA	pBBRMCS2 with the lac promoter deleted and an HA tag inserted	This study
pBBRMCS5	To provide Gm^R for the prey strains in competition assay	Beijing Zoman Biotechnology
pK18-*hcp*1F2R	Suicide vector containing upstream and downstream fragments of *hcp* gene on pK18mob*sacB*; Km^R	This study
pK18-*hcp*AD	Suicide vector containing *hcp* gene with an HA tag fused to the C-terminal of HCP	This study
pK18-*tssm*1F2R	Suicide vector containing upstream and downstream fragments of *tssm* gene on pK18mob*sacB*; Km^R	This study
hcp-HA-pBBR	pBBRNolac containing *hcp* gene with its native promoter and a C-terminal HA tag	This study

Amp^R, Cm^R, Gm^R, and Km^R represent for resistance to ampicillin, chloramphenicol, gentamicin and kanamycin respectively.

Table S2 Primers used in the study

Primer	Description	Sequence（5′-3′）	Source
*hcp*1F	Amplifying the upstream fragments of *hcp*	AGCTATGACATGATTACGGAATTCCGGGAAAGCGGGGCTCTAC	This study
*hcp*1R		GAATCAGAACATATCGACGGACATGGAAT	This study
*hcp*2F	Amplifying the downstream fragments of *hcp*	ATGTTCTGATTCTTCGCTTCGCAAA	This study
*hcp*2R		TGCAGGTCGACTCTAGACGGATCCGGTCGTTCTCGGACCTCTGG	This study
*hcp*HBF	Amplifying the fragments of *hcp* ORF including its native promoter	GGTACCGGGCCCCCCCTCGAGGTAGACGATGCCGAACCCCCCCT	This study
*hcp*HBR		GGCGGCCGCTCTAGAACTAGTGGCTTCTTTGTTTTCCT	This study
*hcp*A	Amplifying the upstream fragment of *hcp* before the stop codon	TGACATGATTACGAATTCATGTCCGTCGATATGTTCA	This study
*hcp*B		TCAAGCGTAGTCTGGGACGTCGTATGGGTAGGCTTCTTTGTTTTCCTTGATGT	This study
*hcp*C	Amplifying the downstream fragment of *hcp* stop codon with an HA tag	GTCCCAGACTACGCTTGATTCTTCGCTTCGCAAAAGCC	This study
*hcp*D		GTCGACTCTAGAGGATTCCGGCAGTGCGGCTTCCTT	This study
*tssm*1F	Amplifying the upstream fragments of *tssm*	TGACATGATTAC GAATTC GCGGAGATGGCCTGT	This study
*tssm*1R		TTGCCAGGACAC GTTTTAGTTCTTCGGATTGC	This study
*tssm*2F	Amplifying the downstream fragments of *tssm*	AAGAACTAAAAC GTGTCCTGGCAATCTCTG	This study
*tssm*2R		GTCGACTCTAGA GGATCC CGAGAAGAACTGCCCGA	This study
*hcp*qF	For detecting *hcp* mRNA in qPCR assay	ATGAAGGTTGAAGGTGCC	This study
*hcp*qR		CGAGTTGGGTTGCGTC	This study
*rpoB*F	For detecting the reference gene *rpoB* mRNA in qPCR assay	GCGACAGCGTGCTCAAAGTG	This study
*rpoB*R		GCCTTCGTTGGTGCGTTTCT	This study
